# Liver Cirrhosis and Septic Shock Attenuate the Increase in Plasma Endostatin Concentrations That Is Associated with Acute Kidney Injury Requiring Dialysis

**DOI:** 10.3390/biomedicines14102142

**Published:** 2026-09-22

**Authors:** Lorena Fröhlich, Caroline Fromm, Lea Läber, Veronika Gepperth, Stephan Schmid, Martina Müller, Patricia Mester, Vlad Pavel, Christa Buechler

**Affiliations:** Department of Internal Medicine I, Gastroenterology, Hepatology, Endocrinology, Rheumatology, and Infectious Diseases, University Hospital Regensburg, 93053 Regensburg, Germany; lorena.froehlich@stud.uni-regensburg.de (L.F.); caroline-anna.fromm@stud.uni-regensburg.de (C.F.); lea.laeber@klinik.uni-regensburg.de (L.L.); veronika.gepperth@klinik.uni-regensburg.de (V.G.); stephan.schmid@klinik.uni-regensburg.de (S.S.); martina.mueller-schilling@klinik.uni-regensburg.de (M.M.); patricia.mester@klinik.uni-regensburg.de (P.M.); vlad.pavel@klinik.uni-regensburg.de (V.P.)

**Keywords:** liver cirrhosis, dialysis, sepsis, COVID-19, endostatin, malaria

## Abstract

**Background/Objectives**: Sepsis is a life-threatening syndrome characterized by a dysregulated host response to infection. Alterations in pro- and anti-angiogenic mediators have been associated with disease severity and clinical outcomes. Endostatin, a potent endogenous inhibitor of angiogenesis, has been linked to renal dysfunction through elevated circulating concentrations. This study investigated whether plasma endostatin levels could serve as a biomarker of acute kidney injury requiring dialysis (AKI-D) and predict disease severity or clinical outcome in patients with sepsis. **Methods**: Plasma endostatin concentrations were quantified by enzyme-linked immunosorbent assay in 258 patients with systemic inflammatory response syndrome (SIRS), sepsis, or septic shock and in 49 healthy controls. Among the patients, 99 had AKI-D, 29 had SARS-CoV-2 infection, 9 had severe malaria, 60 had liver cirrhosis, and 45 had pancreatitis. **Results**: Plasma endostatin concentrations were significantly higher in patients with SIRS, sepsis, or septic shock than in healthy controls. Endostatin levels were further increased in patients with AKI-D and predicted its need with 70% accuracy compared with 72% for the glomerular filtration rate and 72% when both were combined in the non-cirrhosis patients. Subsequent analyses were therefore performed separately in patients with and without AKI-D. In both subgroups, endostatin concentrations were not associated with critical disease severity, survival, or SARS-CoV-2 infection. Liver cirrhosis did not influence endostatin levels in patients not requiring dialysis; however, among patients with AKI-D, cirrhosis attenuated AKI-D-associated upregulation of plasma endostatin. In the subgroup of patients with septic shock, AKI-D was not related to a further increase in plasma endostatin levels. Notably, the nine patients with severe malaria exhibited plasma endostatin levels comparable to those of healthy controls, providing preliminary evidence that the endostatin elevation observed in sepsis was absent in malaria. **Conclusions**: Plasma endostatin concentrations are elevated in patients with SIRS/sepsis/septic shock compared to controls and increase further in AKI-D. However, plasma endostatin levels are not of diagnostic value for prediction of AKI-D, critical illness severity or outcome. The attenuated increase in endostatin levels observed in AKI-D patients with liver cirrhosis or septic shock suggests that circulating endostatin concentrations in AKI-D may be influenced by hepatic function and the severity of critical illness.

## 1. Introduction

Sepsis is a serious condition with a complex pathophysiology that also affects endothelial cells [1,2,3,4]. Endothelial dysfunction is central to inflammatory disorders and contributes to abnormal coagulation, vascular leakage, and further inflammation [5]. Tissue hypoxia and inflammatory cytokines such as tumor necrosis factor increase the expression of vascular endothelial growth factor (VEGF). VEGF stimulates angiogenesis to normalize tissue oxygen supply but also promotes vascular permeability, resulting in tissue edema [4,5]. VEGF has attracted attention as a biomarker in sepsis. A meta-analysis including 20 observational studies that measured systemic VEGF in patients with sepsis found an association of high VEGF with sepsis severity and mortality [6].

Levels of antiangiogenic factors such as angiostatin or thrombospondin have been less well studied in sepsis patients. Notably, serum thrombospondin-1 levels are also increased in sepsis compared with controls [7], indicating that both pro- and antiangiogenic pathways are engaged. In rodent models of sepsis, the loss of thrombospondin-1 protected mice from death, possibly by increasing bacterial clearance [8], indicating that thrombospondin functions beyond angiogenesis.

Endostatin is an endogenous C-terminal fragment of collagen XVIII and a potent angiogenesis inhibitor [9]. Injection of recombinant endostatin dose-dependently increased the survival of septic rodents [10]. Endostatin reduced inflammation and protected mice from organ damage in the liver, lungs, heart, and kidneys [10]. Serum VEGF-A and VEGF-C levels of the septic mice were increased, and endostatin treatment normalized systemic VEGF-C concentration [10]. Endostatin can bind to VEGF receptors, thereby preventing VEGF binding [9,11], and further studies are needed to determine whether its interference with VEGF activity is one of its protective effects in severe inflammation. However, endostatin has also been shown to increase neutrophil activation, migration, and platelet aggregation, all of which are thought to contribute to organ injury in severe COVID-19 [12].

Endostatin is expressed in the kidney, and its levels are induced in renal diseases [13]. Acute renal failure is a common complication of sepsis and can be further compromised by the use of vasopressor therapy [13,14]. Endostatin showed a protective effect in diabetes-related kidney disease [13,15] but promoted renal fibrosis upon endostatin overexpression [16], indicating a disease-related role of endostatin in the kidney.

Early studies showed that endostatin was highly expressed in the liver. Collagen XVIII mRNA was mostly detected in rat hepatocytes and bile duct epithelial cells and occasionally in hepatic stellate cells [17]. Immunoblotting found endostatin in rat and human liver tissues [18,19]. Moreover, ELISA detected endostatin in the supernatant of primary human hepatocytes and hepatic stellate cells [19]. Notably, high endostatin levels in the regenerating rat liver after partial hepatectomy were accompanied by increased endostatin serum levels [18], suggesting that the liver is an important source of circulating endostatin levels.

A recent study reported higher endostatin expression in human subcutaneous adipose tissue than in the liver, but it analyzed fat from only 4 donors and liver tissue from 7 donors [19]. Liver-specific endostatin null mice are not available, and endostatin levels in the hepatic vein of humans have not been evaluated. Therefore, the contribution of the liver, adipose tissue, or other tissues to systemic endostatin levels remains unclear.

The few studies that have measured serum endostatin levels in patients with chronic liver disease have reported inconsistent findings. Several studies observed normal endostatin levels in patients with cirrhosis [20,21], whereas others found higher levels [22,23]. Currently, no study has measured serum endostatin levels in patients with sepsis and cirrhosis.

In patients requiring intensive care, higher plasma endostatin levels were associated with the development of acute kidney injury as well as 30-day mortality [24]. In a study enrolling patients from 17 intensive care units in Finland, higher plasma endostatin levels were observed in patients with acute kidney injury [25]. This study also reported higher plasma endostatin levels in patients with sepsis than in those without sepsis [25]. Notably, a prospective single-center study in patients with sepsis/septic shock observed similar endostatin levels in those with and without acute kidney injury [26]. In this cohort, high plasma endostatin levels on the day acute kidney injury began were associated with renal recovery and improved survival [26].

Serum endostatin was also measured in emergency department patients with COVID-19 and in healthy controls. This study reported that COVID-19 patients had lower serum endostatin levels than the controls, which were not associated with mortality [27]. In contrast, higher plasma endostatin levels were observed in non-surviving COVID-19 patients than in survivors. This study found associations between plasma endostatin and lung function parameters [28]. Higher plasma endostatin levels were also detected in COVID-19 patients than in controls, with no difference in endostatin levels between the 21 COVID-19 and the 21 non-COVID-19 patients, all of whom suffered from acute respiratory distress syndrome (ARDS) [12]. In the COVID-19 ARDS group, levels of the pro-inflammatory acute-phase protein C-reactive protein were weakly and positively related to endostatin concentrations. However, this association was not evident among individuals with non-COVID-19 ARDS, in whom no significant correlation was observed [12].

*Plasmodium falciparum* is responsible for the most serious type of malaria in humans. Although most infected individuals experience only a mild fever-related illness, a proportion of patients develop symptoms and complications associated with severe malaria. In patients with cerebral malaria, researchers have observed an accumulation of collagen XVIII/endostatin in the brain [29]. As organ failure in patients with malaria is associated with endothelial cell apoptosis, which can be induced by endostatin [30], systemic levels of this antiangiogenic molecule may also be elevated, a possibility that has not been tested to our knowledge so far.

This study assessed plasma endostatin concentrations in healthy controls and patients with SIRS/sepsis/septic shock from various causes. The primary objective was to investigate the relationship between circulating endostatin levels and acute renal injury requiring dialysis (AKI-D) in patients with SIRS/sepsis/septic shock. The study also examined whether underlying liver cirrhosis, pancreatitis, severe malaria, and COVID-19 infection were associated with variations in plasma endostatin concentrations.

## 2. Materials and Methods

### 2.1. Study Cohort

Plasma samples were collected from 258 patients admitted to the Medical Intensive Care Unit (ICU) of the University Hospital Regensburg between September 2018 and February 2026. Patients were classified according to the Sepsis-3 criteria [31] and the Systemic Inflammatory Response Syndrome (SIRS) criteria [32] for sepsis, septic shock, and SIRS. We collected plasma samples from COVID-19 patients between October 2020 and October 2024. The nine malaria cases were all *Plasmodium falciparum* infections, and plasma was obtained between August 2023 and September 2025. Patients with human immunodeficiency virus infection were excluded. All other patients who met the inclusion criteria were enrolled in the study.

Blood samples were collected 12–24 h after ICU admission. We added EDTA as an anticoagulant and centrifuged at 3000 rpm to obtain plasma. Blood samples from patients undergoing dialysis were obtained prior to the dialysis procedure. None of the patients had received dialysis before admission to the ICU. Baseline serum creatinine, glomerular filtration rate (GFR), and urea levels at ICU admission are reported in the manuscript. Patients who subsequently required dialysis met the criteria for stage 3 acute kidney injury according to the Kidney Disease: Improving Global Outcomes (KDIGO) classification. Renal replacement therapy was initiated because of septic acute kidney injury accompanied by oligo-anuria. Continuous veno-venous hemodialysis was used in all patients requiring renal replacement therapy. We collected blood samples for endostatin within the first 24 h after ICU admission, while renal replacement therapy was initiated at a median of 3 days after admission.

Patients who died during their ICU stay were classified as non-survivors, whereas those discharged alive were classified as survivors. The Institute of Clinical Chemistry and Laboratory Medicine provided laboratory parameters, and the Institute of Clinical Microbiology and Hygiene at University Hospital Regensburg provided microbiological findings.

Forty-nine healthy donors (25 males and 24 females; *p* = 0.004 compared with the patient cohort; median age, 56 years (21–86 years), *p* = 0.004 compared with the patient cohort) were recruited from among hospital employees, students, and relatives of staff members. All donors were healthy and had a normal body weight. Laboratory parameters were not collected from healthy donors.

### 2.2. Measurement of Plasma Endostatin

The human endostatin ELISA Kit was from Bio-Techne (Wiesbaden-Nordenstadt, Germany, order number: DY1098), and plasma was diluted 1:100 for analysis. We assessed standards and plasma samples twice and used the average values in the calculations.

### 2.3. Statistical Analysis

Endostatin levels in the plasma of patients with SIRS/sepsis/septic shock were not normally distributed (Shapiro–Wilk test, *p* < 0.001). Therefore, data are presented as box-and-whisker plots showing the median, first and third quartiles, and minimum and maximum values, with asterisks or circles indicating outliers. Median, minimum, and maximum values for each dataset are also provided in the tables. Statistical analyses were performed using IBM SPSS Statistics 31.0.0.0 (IBM Corp., Armonk, NY, USA; released 2019, updated 2025). The following tests were used to assess relationships between variables:

1. Chi-squared test for categorical variables;
2. Kruskal–Wallis test for comparison of three or more groups;
3. Mann–Whitney U test for comparisons between two groups;
4. Multiple linear regression;
5. Binary logistic regression;
6. Receiver operating characteristic (ROC) curve analysis;
7. Spearman’s correlation for associations between continuous variables;
8. G*Power 3.1.6 for power analysis [33].

A two-sided *p*-value < 0.05 was considered statistically significant.

We did not correct the results for multiple comparisons for the reasons discussed elsewhere [34,35]. We defined the statistical analyses in advance based on the hypothesis that AKI-D could influence endostatin concentrations in patients with SIRS, sepsis, or septic shock. Thus, the study was not designed to search for statistically significant relationships among a broad range of potential variables or outcomes. Because only a limited number of clinically related hypotheses were prespecified, we did not consider multiple-comparison correction appropriate, as this would constitute a post hoc statistical adjustment rather than an a priori component of the analysis plan.

## 3. Results

### 3.1. Endostatin in Plasma of Controls and Patients

Plasma endostatin levels were measured in 49 controls and 258 patients with SIRS/sepsis/septic shock. Patients with SIRS/sepsis/septic shock had higher plasma endostatin levels (183 (19–1609) ng/mL vs. 97 (40–200) ng/mL) than controls (Figure 1a). The area under the receiver operating characteristic curve (AUROC) was 0.821 ± 0.025 (95% CI 0.773–0.870; *p* < 0.001) for diagnosing SIRS/sepsis/septic shock, and 137 ng/mL endostatin had a sensitivity of 69% and a specificity of 88%. Thus, although plasma endostatin is not a stand-alone diagnostic test for sepsis, its consistent elevation reflects the septic host response and can complement established markers.

Among the controls, there were more females, and a sex-specific analysis was also done. The 188 male patients had higher plasma endostatin levels than the 25 male controls (*p* < 0.001). Age differed significantly (*p* = 0.033). The 70 female patients had higher plasma endostatin levels than the 24 female controls (*p* < 0.001). Age differed significantly (*p* = 0.009).

An age-matched analysis was also done in patients aged above 58 years, where age did not differ significantly between patients and controls (*p* = 0.067). In the 148 patients, endostatin was higher (*p* < 0.001) than in the 15 controls. To assess the statistical strength of this finding, we performed a post hoc power analysis for the two-group comparison. With an alpha level of 0.05 and the observed effect size of 0.99, the evaluation yielded a statistical power of 97%.

Across the entire cohort, the 58 patients with SIRS, the 67 with sepsis, and the 133 with septic shock had similar plasma endostatin levels (Figure 1b).

Because previous studies reported higher endostatin levels in patients with renal disease [13,25], the association between plasma endostatin levels and the need for dialysis was first analyzed.

Most patients with AKI-D belonged to the sepsis or septic shock group (Table 1). Procalcitonin, aspartate aminotransferase, bilirubin, creatinine, and urea were higher, and glomerular filtration rate (GFR) was lower in AKI-D patients (Table 1).

Indeed, plasma endostatin levels in the 99 AKI-D patients were significantly higher than in patients who did not subsequently require dialysis (Figure 1c).

Multiple linear regression showed that age, sex, bilirubin, GFR, aspartate aminotransferase, procalcitonin, disease severity (SIRS, sepsis, septic shock), and need for dialysis significantly predicted plasma endostatin levels (F (8.211) = 3.927, *p* < 0.001). GFR (*p* < 0.001), bilirubin (*p* = 0.018), and dialysis (*p* = 0.028) were significant.

The AUROC for endostatin in predicting dialysis was 0.644 ± 0.034 (95% CI 0.577–0.712; *p* < 0.001), indicating acceptable discriminative performance for identifying patients with AKI-D. Binary logistic regression showed that plasma endostatin correctly classified the need for dialysis in 62% of patients; combining it with GFR improved classification to 72%, compared with 71% for GFR alone. The baseline model, which included none of these predictors, correctly classified 62%. These analyses suggest that plasma endostatin is not diagnostically useful for predicting AKI-D in SIRS/sepsis/septic shock.

Because endostatin levels were higher in patients with AKI-D (Figure 1c), we analyzed patients with and without dialysis separately.

### 3.2. Endostatin in the Plasma of Patients Without Dialysis

In patients without AKI-D, 117 males and 42 females had similar plasma endostatin levels (*p* = 0.272), and we analyzed both sexes together.

After excluding patients with AKI-D, plasma endostatin levels remained higher than in controls (Figure 2a). This was significant in males (117 cases and 25 controls; *p* < 0.001) with a modest age difference between these cohorts (*p* = 0.047) and in females (42 cases and 24 controls; *p* < 0.001 for endostatin and *p* = 0.005 for age).

Patients with urea > 50 mg/dL (96 patients) had higher plasma endostatin levels than those with urea < 50 mg/dL (63 patients) (Figure 2b). The 63 patients with creatinine < 1 mg/dL had lower plasma endostatin levels than patients with creatinine > 1 mg/dL (82 patients) (Figure 2c). In the patients not requiring dialysis, plasma endostatin levels increased with lower GFR (Figure 2d). GFR (mL/min/1.73 m^2^) was >90 in 46 patients, 60–89 in 30 patients, 45–59 in 25 patients, 30–44 in 20 patients, 15–29 in 22 patients, and <15 in 5 patients.

Further interventions included ventilation and vasopressor therapy. However, the 86 patients requiring ventilation (*p* = 0.712) and the 93 patients requiring vasopressor therapy (*p* = 0.925) had plasma endostatin levels similar to those of patients without these interventions.

In the non-dialysis cohort, endostatin levels did not differ significantly among the 51 patients with SIRS, the 55 with sepsis, and the 53 with septic shock (*p* = 0.054). Moreover, endostatin levels did not differ between survivors and non-survivors (17 patients, *p* = 0.157; Figure 3a).

Underlying diseases such as liver cirrhosis may be associated with altered systemic endostatin levels [9]. In the subcohort not requiring dialysis, 30 patients had liver cirrhosis, and 32 had pancreatitis. Patients with cirrhosis (*p* = 0.181) or pancreatitis (*p* = 0.061) had plasma endostatin levels similar to those without cirrhosis or pancreatitis (Figure 3b). The Child–Pugh score was documented for 29 patients (score A: 3 patients, B: 11 patients and C: 15 patients) but was not related to plasma endostatin levels (*p* = 0.345).

The 18 patients with COVID-19 had plasma endostatin levels similar to those of non-COVID-19 patients (*p* = 0.347).

In the SIRS/sepsis/septic shock cohort, 9 patients had malaria (6 had SIRS, 2 had sepsis, and 1 had septic shock). Interestingly, these patients had lower plasma endostatin (107 (44–164) ng/mL) than patients with other sepsis etiologies (171 (19–581) ng/mL), and levels were similar to those of the healthy controls (97 (40–200) ng/mL) (Figure 3c). To assess the statistical strength of this finding, we performed a post hoc power analysis for the two-group comparison (patients with malaria compared to patients with a different disease etiology). With an alpha level of 0.05 and the observed effect size of 1.018, the evaluation yielded a statistical power of 83%.

Patients with malaria were younger (*p* = 0.007) and had fewer leukocytes (*p* = 0.027) and neutrophils (*p* = 0.023) than patients with SIRS/sepsis/septic shock without malaria.

### 3.3. Endostatin in the Plasma of Patients with Dialysis

In the subcohort of 99 patients requiring dialysis, endostatin levels did not differ by sex. The 30 patients with liver cirrhosis had greatly reduced serum endostatin levels, at 258 (71–1609) ng/mL in patients without cirrhosis and 168 (78–366) ng/mL in patients with cirrhosis (*p* < 0.001; Figure 4a). Plasma endostatin levels in patients with cirrhosis requiring dialysis remained higher than those in healthy controls (*p* < 0.001).

The Child–Pugh score was A: 2 patients, B: 13 patients and C: 15 patients and was not related to plasma endostatin levels (*p* = 0.127).

Comparison of cirrhosis patients with and without AKI-D revealed that, in this subgroup, the subsequent need for dialysis was not associated with higher plasma endostatin levels (Figure 4b).

Patients on dialysis with cirrhosis had lower age (*p* = 0.004), lower C-reactive protein (*p* < 0.001), higher monocyte number (*p* = 0.012), higher bilirubin (*p* < 0.001) and albumin (*p* = 0.006), and lower gamma-glutamyltransferase (*p* = 0.021) compared with non-cirrhosis AKI-D patients. Creatinine, urea, and GFR did not differ between AKI-D patients with and without cirrhosis, suggesting similar renal function of the cohorts.

AKI-D patients with creatinine < 1 mg/dL (11 patients) and those with higher levels (75 patients) had similar endostatin levels (*p* = 0.323). The 20 patients with urea < 50 mg/dL had modestly higher endostatin than the 79 patients with levels above 50 mg/dL (*p* = 0.033). In the patients requiring dialysis, plasma endostatin levels did not change with lower GFR (*p* = 0.158). GFR (mL/min/1.73 m^2^) was >90 in 9 patients, 60–89 in 10 patients, 45–59 in 8 patients, 30–44 in 13 patients, 15–29 in 28 patients, and <15 in 21 patients.

Binary logistic regression showed that, in the entire cohort, plasma endostatin correctly classified AKI-D in 70% of patients when excluding those with liver cirrhosis. For endostatin and GFR, the rate was 72%, and GFR alone also classified 72% of patients correctly. The baseline model, which included none of these predictors, classified 65% correctly.

Excluding those with liver cirrhosis, the 49 males and 20 females had similar plasma endostatin levels (*p* = 0.561). Pancreatitis in 11 patients was not associated with altered plasma endostatin levels (*p* = 0.635). The 9 patients with COVID-19 had plasma endostatin levels similar to those of non-COVID-19 patients (*p* = 0.762). Endostatin levels did not differ between survivors and non-survivors (36 survivors and 33 non-survivors; *p* = 0.116).

Sixty-one patients needed ventilation, and plasma endostatin levels did not differ (*p* = 0.807). This also applied to the 66 patients requiring vasopressor therapy (*p* = 0.312). However, the number of patients with pancreatitis or COVID-19 and those without these interventions is small, limiting the statistical power of these comparisons.

### 3.4. Correlation Analysis

Correlation analysis was done separately in patients with and without the need for dialysis. In both subgroups, plasma endostatin was negatively correlated with GFR and bilirubin, and positively correlated with urea and creatinine (Table 2). Plasma endostatin was positively correlated with C-reactive protein in non-dialysis patients and negatively correlated with monocyte count in dialysis patients (Table 2).

Excluding patients with liver cirrhosis from the AKI-D cohort the negative correlation of endostatin with bilirubin (r = −0.327, *p* = 0.007) and GFR (r = −0.314, *p* = 0.012) and the positive correlation with creatinine (r = 0.305, *p* = 0.012) and urea (r = 0.310, *p* = 0.010) were still significant.

### 3.5. Separate Analysis in Sepsis and Septic Shock

Patients with AKI-D more often had sepsis or septic shock (Table 1), and endostatin levels were analyzed separately in these subcohorts. The 12 AKI-D patients in the sepsis cohort had higher plasma endostatin than the 55 sepsis patients without this complication (*p* < 0.001). Creatinine (*p* < 0.001), urea (*p* < 0.001), and gamma glutamyltransferase (*p* < 0.001) were also higher and GFR (*p* < 0.001) was reduced.

The 80 septic shock patients with AKI-D had endostatin levels similar to those of the 53 non-AKI-D patients, but higher procalcitonin (*p* = 0.006), bilirubin (*p* < 0.001), creatinine (*p* < 0.001) and AST (*p* = 0.012) and lower GFR (*p* < 0.001), than septic shock patients who did not require dialysis. Similar results were obtained when excluding the 25 patients with liver cirrhosis. This subgroup analysis suggests that the association of endostatin levels with AKI-D is attenuated in patients with septic shock.

However, in the entire cohort, the prevalence of SIRS, sepsis, and septic shock among patients with and without cirrhosis was similar (*p* = 0.150), arguing against higher disease severity as the cause of unchanged plasma AKI-D in cirrhosis.

## 4. Discussion

This study demonstrated that plasma endostatin concentrations were elevated in patients with SIRS/sepsis/septic shock and were even higher in patients with AKI-D. Notably, the higher plasma endostatin associated with AKI-D was attenuated in patients with liver cirrhosis, suggesting that liver dysfunction affects plasma endostatin levels in AKI-D patients. Furthermore, the association between AKI-D and plasma endostatin concentrations was attenuated in patients with septic shock, indicating that the severity of critical illness may also influence circulating endostatin levels.

One hypothesis is that endostatin is higher in AKI-D to protect from increased angiogenesis, and a blunted response in cirrhosis and septic shock may contribute to increased disease severity [36,37]. However, this is an observational study and cannot provide proof for this suggestion.

In critically ill patients, higher levels of angiogenic factors such as VEGF have been consistently described [6]. However, antiangiogenic factors have received less attention. It has been shown that soluble tyrosine kinase 1 type fms, which hinders binding of VEGF to its receptor, is induced in sepsis [38]. Angiostatin was increased in the plasma of COVID-19 patients with moderate and severe disease [39]. Serum thrombospondin-1, which also hinders VEGF signaling, is increased in sepsis compared with controls [7]. To the best of our knowledge, no studies have examined these angiogenic proteins in patients with sepsis and AKI-D.

Only a few studies previously analyzed endostatin levels in critically ill patients [25,26,28,40]. However, a detailed subcohort analysis has never been performed. In the present study, we measured endostatin in the entire SIRS/sepsis/septic shock cohort and compared various aspects of septic patients, including vasopressor therapy, invasive ventilation, and sepsis etiologies such as viral and parasitic diseases.

In our cohort, plasma endostatin was not an effective marker for predicting AKI-D and was no better than routine diagnostic measures. Faris et al. showed that plasma endostatin levels at admission were similar in 25 sepsis patients with AKI and 15 patients without AKI [26]. However, this cohort was comparatively small, which is a limitation of this study [26]. Ruge et al. used the same ELISA and analyzed EDTA plasma [24] as we did. The median endostatin levels were 38 ng/L in patients without AKI and 87 ng/mL in AKI-D, respectively [24], compared to 200 ng/mL in non-AKI-D and 300 ng/mL in AKI-D patients in our study groups. The AKI-D patients included in the study by Ruge et al. had a median creatinine level of 1.5 mg/dL, which was lower than that of our patients (median 2.3 mg/dL). The simplified Acute Physiology Score 3 (SAPS3) was higher in patients with renal disease in the study by Ruge et al. [24], and our patients with AKI-D also had more severe disease. As in our cohort, blood samples were collected at ICU admission, and patients who developed AKI within 7 days (about 3 days in our cohort) had higher plasma endostatin, which increased further in those on renal replacement therapy, although this procedure was not described in more detail [24]. The study by Ruge et al. [24] and the current findings suggest that systemic endostatin is associated with renal failure but may not serve as a stand-alone prognostic tool.

GFR significantly predicted plasma endostatin levels and was inversely correlated with endostatin concentrations. The markedly reduced GFR in patients undergoing dialysis indicates that renal dysfunction is the primary determinant of elevated plasma endostatin levels in patients with SIRS/sepsis/septic shock. This interpretation is further supported by the positive correlations observed between plasma endostatin and both urea and creatinine concentrations. Endostatin correlations with measures of renal function were highly significant in non-AKI-D patients. Here, plasma endostatin levels declined with lower GFR and increased with higher urea and creatinine levels. These associations were weak or absent in the AKI-D cohort, with modest effect sizes and high *p*-values in the correlation analysis.

Notably, in septic shock, plasma endostatin levels were similar between patients with and without AKI-D, although the latter cohort had lower GFR and higher creatinine levels. In sepsis patients, AKI-D was related to higher plasma endostatin, showing that the attenuated response is specific to septic shock. Therefore, this study suggests that renal disease and critical illness severity both affect plasma endostatin levels.

The current study did not analyze urinary endostatin. In patients with diabetic kidney disease, urinary endostatin was increased [41], indicating that renal disease does not account for the higher plasma levels by reducing urinary excretion. Immunohistochemistry showed elevated endostatin levels in renal tubular cells from patients with diabetic kidney disease, indicating increased release from this organ [41]. However, immunostaining cannot distinguish collagen XVIII from endostatin, and this analysis cannot resolve whether more of the latter is produced [41].

Liver cirrhosis was a relatively common underlying disease in our cohort. Comparison of patients with liver cirrhosis who were and were not on dialysis showed that, among patients with cirrhosis, dialysis was not associated with higher plasma endostatin levels, in contrast to what was observed in the entire cohort. In the non-dialysis group, liver cirrhosis was not associated with changed endostatin levels. Various proteins produced in the liver, such as C-reactive protein and lipopolysaccharide-binding protein, are low in the circulation of patients with liver cirrhosis, whereas proteins cleared by the liver, such as interleukin-6, are induced [42,43,44]. This primarily argues against the liver as a major contributor to systemic endostatin levels in patients with SIRS/sepsis/septic shock, or at least suggests that liver cirrhosis is not associated with lower endostatin levels.

A comparison of cirrhosis and non-cirrhosis patients with sepsis showed that markers of endothelial activation, such as angiopoietin-2 and soluble thrombomodulin, are higher in cirrhosis at ICU admission [45]. Plasma endostatin levels differ between cirrhosis and non-cirrhosis AKI-D patients, and endothelial function may be affected differently in these cohorts.

Conventional biomarkers used to evaluate renal function may be unreliable in patients with liver cirrhosis. Reduced hepatic creatinine production, together with increased renal tubular secretion of creatinine, can lead to falsely low serum creatinine concentrations. Consequently, creatinine-based methods may overestimate the patient’s actual glomerular filtration rate [46]. As endostatin does not change with renal failure in cirrhosis, it is not a marker of kidney disease in these patients.

Interestingly, patients with severe malaria did not have elevated plasma endostatin levels; these were within the normal range. Severe malaria is associated with endothelial dysfunction and the upregulation of proangiogenic molecules such as VEGF [47]. An in vitro study suggested that similar mechanisms contribute to endothelial apoptosis in malaria and bacterial sepsis [48]. Systemic endothelial activation in malaria and sepsis was comparable, with upregulation of endothelial inducible cell adhesion molecules and higher levels of soluble cell adhesion molecules, as well as tumor necrosis factor, observed in both cohorts [49]. In children with malaria, inflammatory responses were higher than in sepsis, while children with sepsis had a higher pro-inflammatory-to-anti-inflammatory ratio [50]. Unchanged plasma endostatin levels in malaria-associated critical illness, compared with the strong increases in sepsis patients across different disease etiologies, is another difference between these patients whose pathological and clinical relevance needs further study. Higher collagen XVIII/endostatin protein levels have been observed in malaria patients’ brains [29]. Endostatin does not pass the blood–brain barrier [51], and systemic levels may not correlate with central concentrations. The finding that severe inflammatory diseases such as malaria are associated with normal plasma endostatin levels awaits confirmation in larger cohorts, as only nine patients have been included. However, these findings may suggest that endostatin has no or a minor role in the pathophysiological pathways of parasitic infections.

Our analysis also confirms previous studies showing higher plasma endostatin levels in patients with SIRS/sepsis/septic shock, with similar elevations in both COVID-19 and non-COVID-19 sepsis compared with healthy controls [12,25]. Patients with SIRS, sepsis, and septic shock had similar plasma endostatin levels, which were also not associated with survival. Akdur et al. reported that COVID-19 patients in the emergency department who did not require intensive care had lower serum endostatin levels than the controls [27]. Asif et al. enrolled severely ill COVID-19 patients requiring intensive care but did not include healthy controls [28]. In that study, 56% of the patients developed AKI, but whether dialysis was required was not discussed [28]. This study reported higher endostatin in 30-day non-survivors, with a mortality of 25%, similar to our mortality rate of 27%. Higher systemic endostatin may thus be a feature of critical illness and may not increase in patients with mild or moderate COVID-19 [27]. However, discordant findings on the association between endostatin and mortality remain unclear.

Our study has several limitations. First, its single-center design may limit the generalizability of the findings. Second, dialysis was used as a surrogate for acute kidney injury, without staging, potentially resulting in misclassification and limiting comparisons with other studies. Third, the malaria subgroup was very small (*n* = 9), substantially limiting the strength and generalizability of conclusions regarding malaria-associated findings. Fourth, the cohort included heterogeneous sepsis etiologies, which may have introduced clinical and biological heterogeneity. Fifth, the cirrhosis comparison may remain subject to residual confounding, particularly by differences in age and underlying liver disease characteristics. Sixth, plasma endostatin concentrations were measured only once at ICU admission, preventing evaluation at symptom onset and longitudinal changes throughout the clinical course. Consequently, we could not determine the prognostic significance of dynamic fluctuations in endostatin levels. Moreover, urine samples were not collected concurrently with blood samples; therefore, urinary endostatin measurements were unavailable for analysis. A key strength of this study is the comparatively large patient cohort, which allows for meaningful analysis and separate assessment of distinct subgroups. Because this study did not correct for multiple comparisons, significant findings, such as the comparison between patients with and without malaria, could be due to chance.

## 5. Conclusions

Overall, plasma endostatin concentrations were consistently higher in patients with SIRS/sepsis/septic shock and were further increased in those patients with AKI-D. Endostatin alone was not more effective than established renal markers as a standalone diagnostic test for AKI-D. Notably, in patients with AKI-D and concomitant liver cirrhosis, plasma endostatin levels were not higher, demonstrating that liver function significantly impacts circulating endostatin levels during septic renal failure. Endostatin levels are similar between non-AKI-D and AKI-D patients with septic shock, showing that severe critical illness also affects endostatin levels in renal failure.

## Figures and Tables

**Figure 1:**
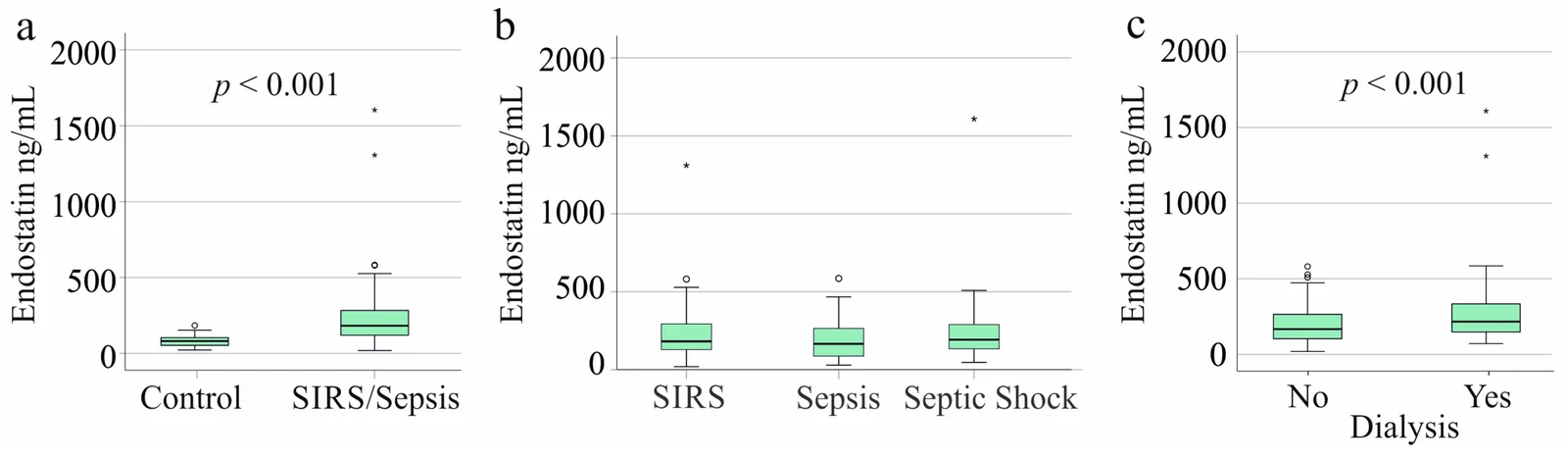
Endostatin in the plasma of controls, patients with systemic inflammatory response syndrome (SIRS), sepsis, or septic shock, and the effect of dialysis. (**a**) Endostatin in the plasma of controls and patients; (**b**) endostatin in the plasma of patients with SIRS, sepsis, or septic shock; (**c**) endostatin in the plasma of patients without (No) and with (Yes) subsequent dialysis need. Outliers are represented by circles and small asterisks.

**Figure 2:**
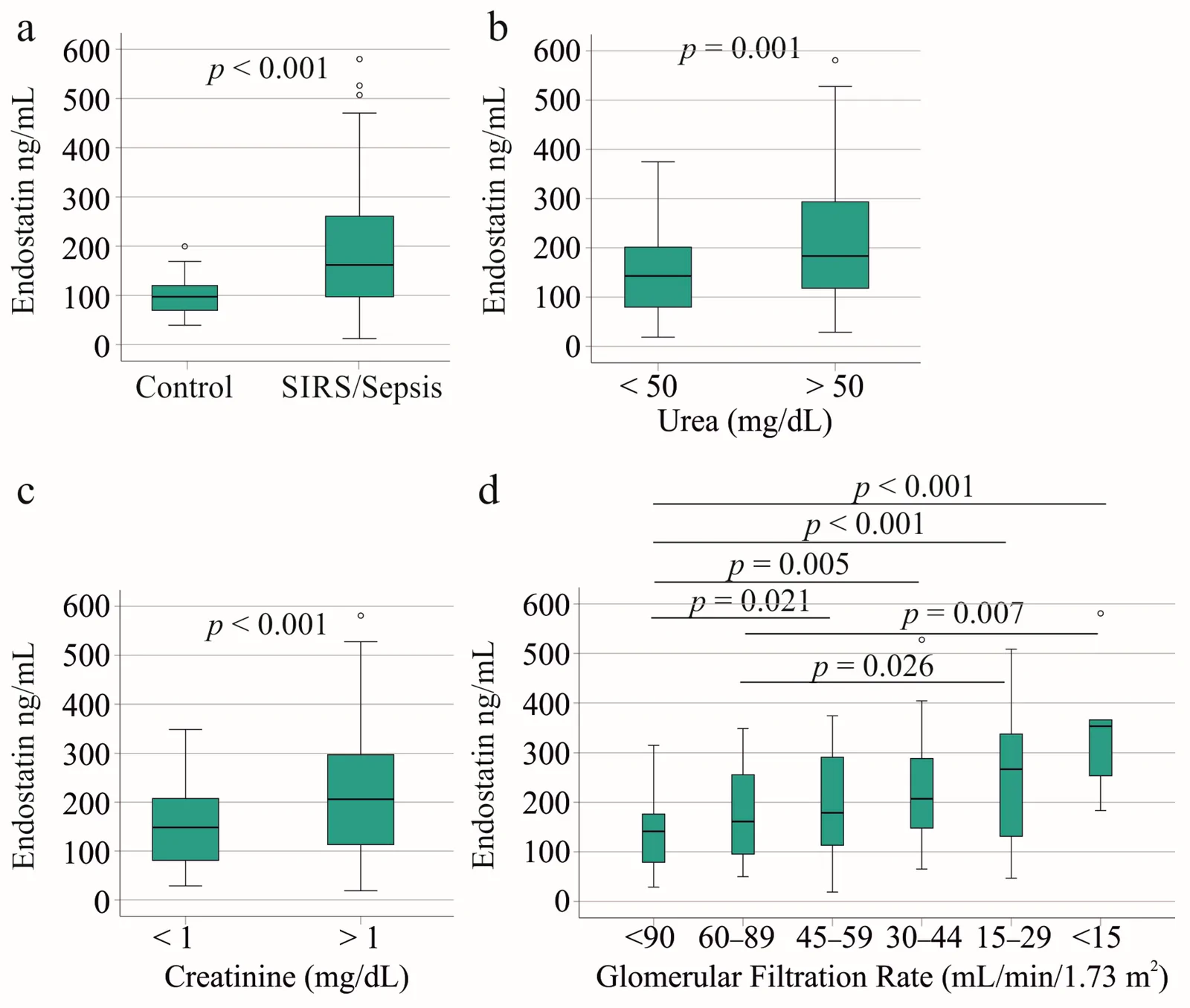
Endostatin in the plasma of controls and patients not requiring dialysis and its relation to renal function. (**a**) Endostatin in the plasma of controls and patients not requiring dialysis; (**b**) plasma endostatin levels of the patients with urea below and above 50 mg/dL; (**c**) plasma endostatin levels of patients with creatinine below and above 1 mg/dL; (**d**) plasma endostatin levels of the patients stratified for glomerular filtration rate. Outliers are represented by small circles.

**Figure 3:**
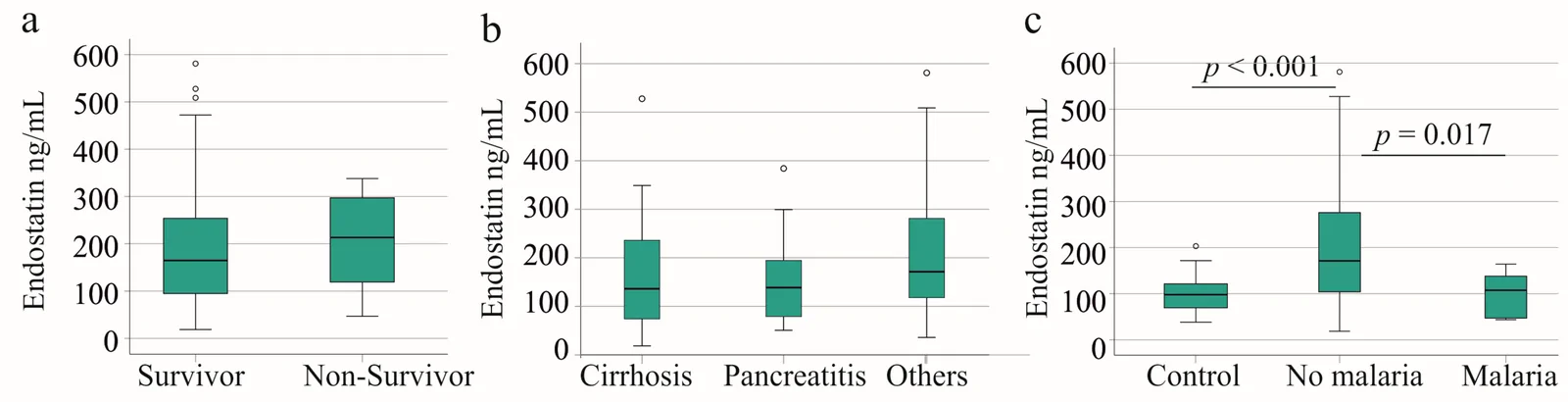
Endostatin in the plasma of patients not requiring dialysis. (**a**) Endostatin in survivors and non-survivors; (**b**) plasma endostatin levels of SIRS/sepsis/septic shock patients with cirrhosis or pancreatitis compared to patients without these underlying diseases; (**c**) plasma endostatin levels of controls and SIRS/sepsis patients with and without malaria. Outliers are represented by small circles.

**Figure 4:**
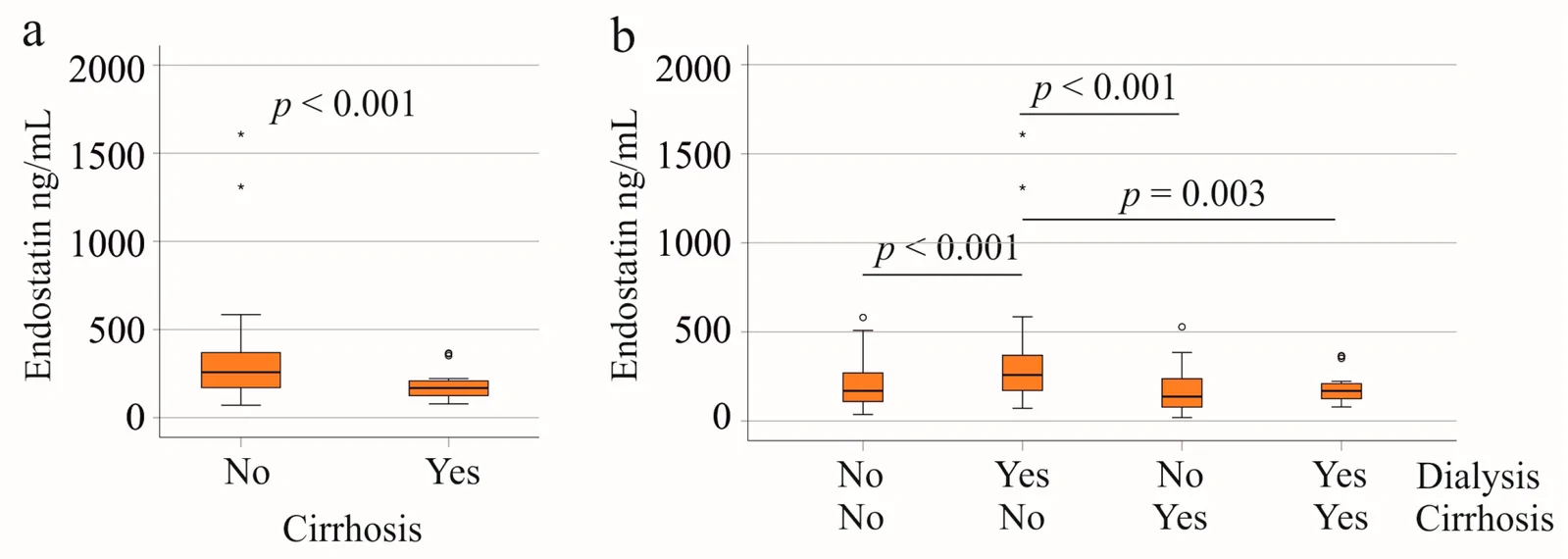
Endostatin in relation to cirrhosis and dialysis. (**a**) Endostatin in the plasma of patients without (No) and with (Yes) liver cirrhosis among those patients requiring dialysis; (**b**) plasma endostatin of patients with and without AKI-D with and without liver cirrhosis. Outliers are represented by small circles and asterisks.

**Table 1:** Characteristics of SIRS/sepsis/septic shock patients without (−) and with (+) AKI-D. Blood samples were obtained before dialysis. Data are presented as medians, minimums, and maximums. Superscript numbers indicate that data were not available for the entire cohort. Statistical tests used: Mann–Whitney U test and chi-square test.

Parameters	Patients Without AKI-D (159)	Patients with AKI-D (99)	*p*-Value
Males/Females	117/42	71/28	Not significant
Age (years)	61 (17–93)	59 (21–86)	Not significant
Body Mass Index (kg/m^2^)	26.5 (13.2–60.2)	27.8 (17.5–51.0)	Not significant
SIRS/Sepsis/Septic Shock	51/55/53	7/12/80	<0.001
C-reactive protein mg/L	146 (6–697)	131 (2–518)	Not significant
Procalcitonin ng/mL	1.36 (0.05–497.00)	3.41 (0.18–120.00)	0.002
Leukocytes n/nL	10.21 (0.06–246.94)	12.61 (0.01–57.91)	Not significant
Neutrophils n/nL	8.50 (0–72.59)	10.66 (0–50.61)	Not significant
Basophils n/nL	0.03 (0–3.20)	0.04 (0–0.52)	Not significant
Eosinophils n/nL	0.03 (0–8.80)	0.02 (0–0.94)	Not significant
Monocytes n/nL	0.64 (0–45.00)	0.74 (0–14.80)	Not significant
Lymphocytes n/nL	0.80 (0.01 –28.60)	0.77 (0–100.00)	Not significant
Immature Granulocytes n/nL	0.17 (0–6.19)	0.24 (0–7.25)	Not significant
Aspartate aminotransferase U/L	48 (7–1703) ^149^	74 (13–8174) ^96^	<0.001
Alanine aminotransferase U/L	32 (5–1139) ^142^	49 (5–2360) ^96^	Not significant
Albumin g/L	28.0 (15.9–61.3) ^147^	28.0 (6.3–43.6) ^96^	Not significant
Gamma glutamyltransferase U/L	122 (11–1923) ^144^	126 (11–1280) ^90^	Not significant
Total Bilirubin mg/dL	0.90 (0.20–33.90) ^151^	2.40 (0.20–26.80) ^97^	<0.001
Direct Bilirubin mg/dL	0.60 (0.10–22.50) ^151^	1.82 (0.10–19.20) ^93^	<0.001
Indirect Bilirubin mg/dL	0.30 (0.05–11.40) ^141^	0.75 (0.07–8.10) ^88^	<0.001
Urea mg/dL	57 (8–232)	92 (8–347)	<0.001
Creatinine mg/dL	1.1 (0.3–8.9) ^145^	2.3 (0.3–16.1) ^86^	<0.001
Glomerular filtration rate mL/min/1.73 m^2^	62 (5–174) ^148^	28 (3–134) ^89^	<0.001

**Table 2:** Spearman correlation coefficients in patients without acute kidney injury requiring dialysis (AKI-D) and with the need for dialysis.

Biomarkers	Patients Without AKI-D		Patients with AKI-D	
	r	*p*-value	r	*p*-value
Age (years)	0.145	Not significant	0.045	Not significant
Body Mass Index (kg/m^2^)	0.023	Not significant	0.166	Not significant
C-reactive protein mg/L	0.201	0.011	0.059	Not significant
Procalcitonin ng/mL	0.143	Not significant	0.073	Not significant
Leukocytes n/nL	0.149	Not significant	0.005	Not significant
Neutrophils n/nL	0.142	Not significant	−0.079	Not significant
Basophils n/nL	−0.052	Not significant	−0.047	Not significant
Eosinophils n/nL	−0.012	Not significant	−0.039	Not significant
Monocytes n/nL	−0.032	Not significant	−0.206	0.041
Lymphocytes n/nL	0.002	Not significant	0.016	Not significant
Immature Granulocytes n/nL	0.010	Not significant	0.195	Not significant
Urea mg/dL	0.293	<0.001	0.223	0.029
Creatinine mg/dL	0.371	<0.001	0.243	0.024
Glomerular filtration rate mL/min/1.73 m^2^	−0.406	<0.001	−0.256	0.015
Aspartate aminotransferase U/L	−0.084	Not significant	−0.116	Not significant
Alanine aminotransferase U/L	−0.123	Not significant	−0.027	Not significant
Albumin g/L	−0.096	Not significant	−0.011	Not significant
Gamma glutamyltransferase U/L	0.000	Not significant	0.110	Not significant
Total Bilirubin mg/dL	−0.171	0.036	−0.347	0.001
Direct Bilirubin mg/dL	−0.141	Not significant	−0.354	<0.001
Indirect Bilirubin mg/dL	−0.190	0.024	−0.373	<0.001

## Data Availability

The raw data supporting the conclusions of this article will be made available by the authors on request.

## Abbreviations

AKI-D	Acute kidney injury requiring dialysis
ALT	Alanine aminotransferase
ARDS	Acute respiratory distress syndrome
AST	Aspartate aminotransferase
CRP	C-reactive protein
GFR	Glomerular filtration rate
GGT	Gamma-glutamyl transferase
ICU	Intensive care unit
ROC	Receiver operating characteristic
SARS-CoV-2	Severe acute respiratory syndrome coronavirus 2
SIRS	Systemic inflammatory response syndrome
VEGF	Vascular endothelial growth factor
